# Supplementary material for: Structural basis for phage-mediated activation and repression of bacterial DSR2 anti-phage defense system
Source: Nat Commun. 2024 Mar 30;15:2797. doi: 10.1038/s41467-024-47177-9 (PMC10981675; doi:10.1038/s41467-024-47177-9)

Supplementary Figures and Table

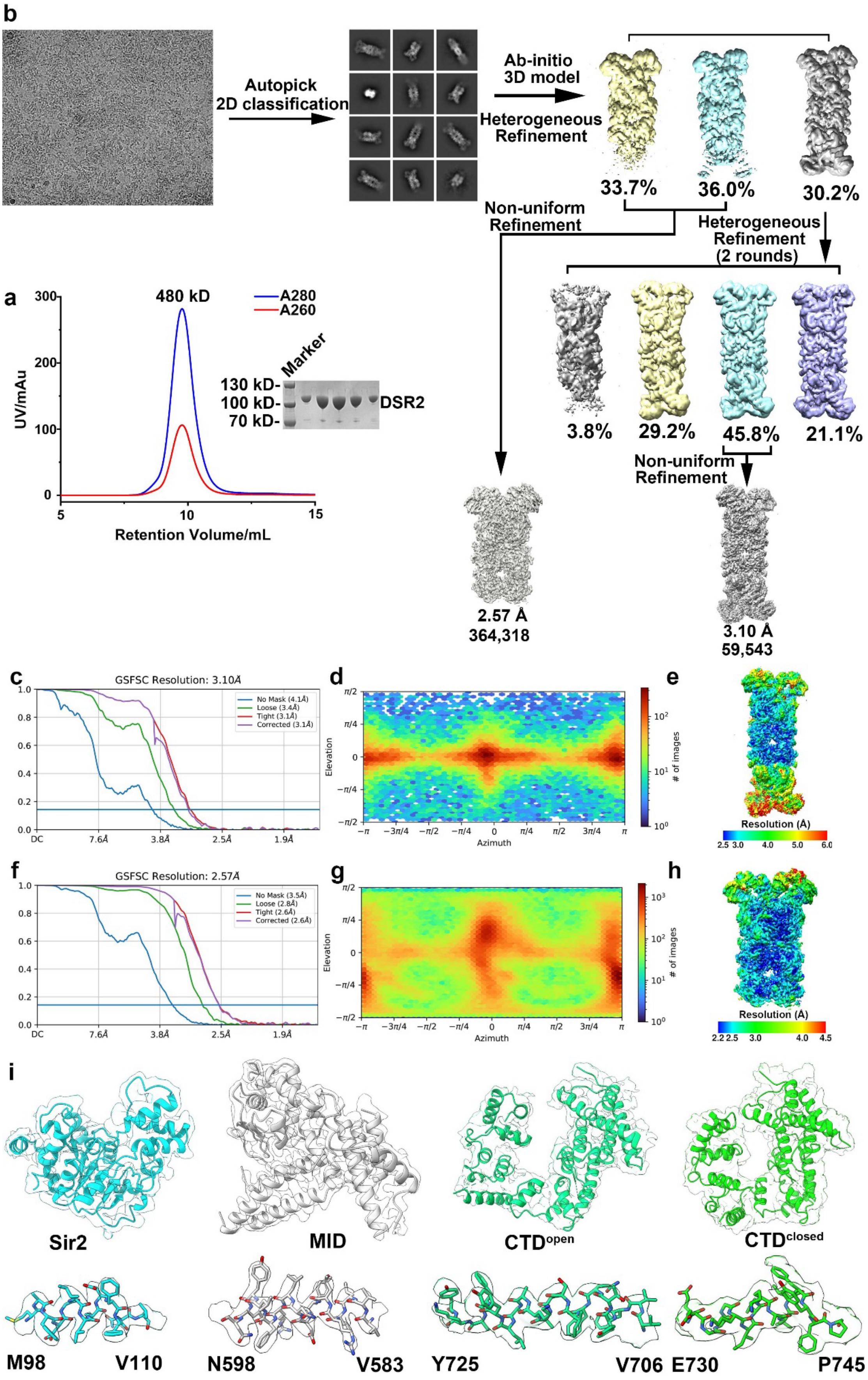

Supplementary Fig. 1: Cryo-EM reconstruction of the apo form of DSR2.

a, Size exclusion chromatography and SDS-PAGE profiles of the purified apo form of DSR2. **b,** Flow chart of image processing for apo DSR2. **c, f,** Fourier Shell Correlation curve of tetrameric DSR2. **d, g,** Direction distribution plot of tetrameric DSR2. **e, h,** Final 3D reconstructed map of tetrameric DSR2, colored according to local resolution. **i,** Cryo-EM densities of each domain of apo DSR2. Densities for the indicated regions are shown in the context of the atomic model. Source data are provided as a Source Data file.

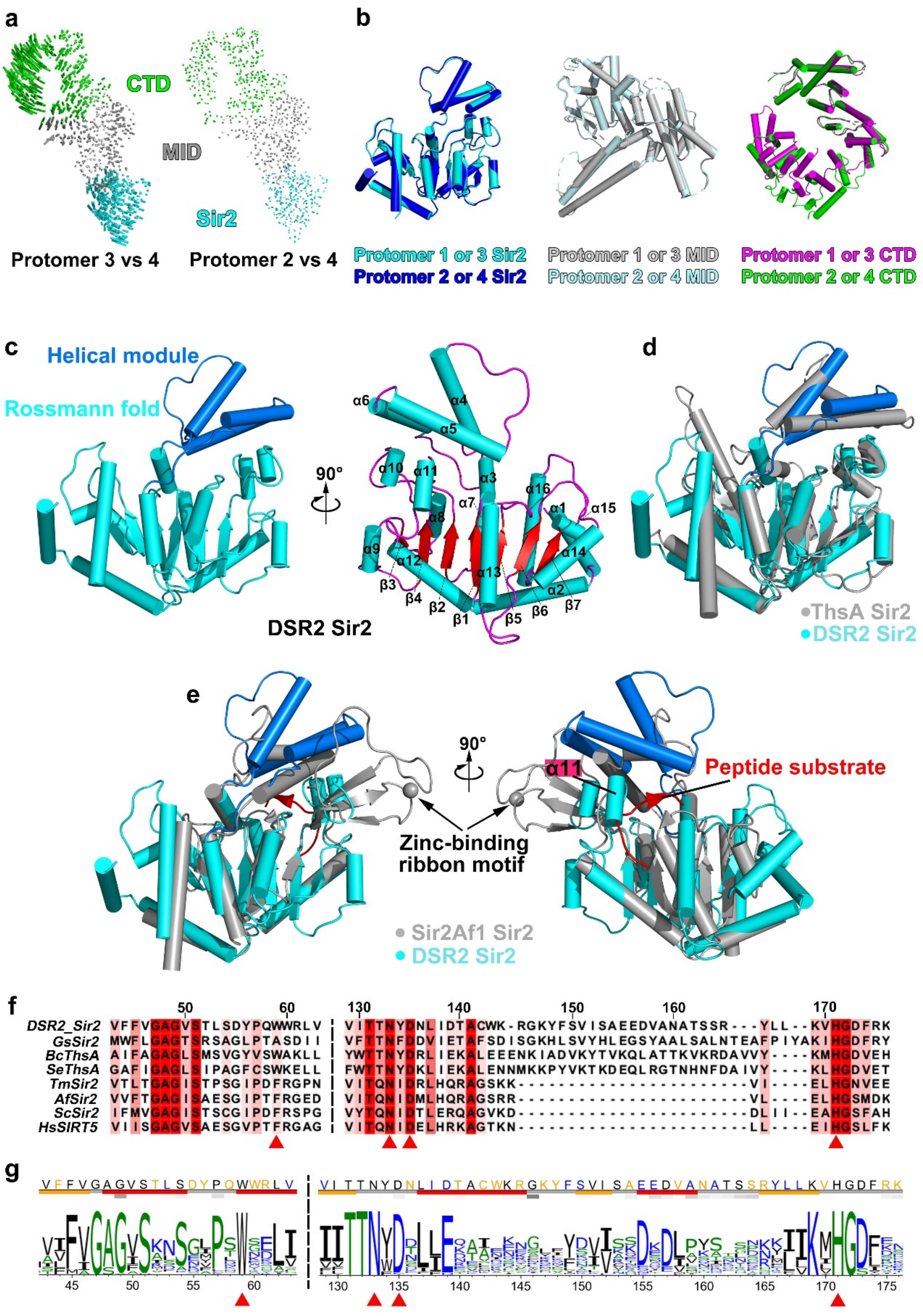

Supplementary Fig. 2: Structure and sequence comparisons of the DSR2 with related proteins.

a, Structural comparison of protomer 3 compared to protomer 4 and protomer 2 relative to protomer 4. Vector length correlates with the scale of domain movement. **b,** Structural comparison of the Sir2 domain, MID and CTD located in protomer 1 or

protomer 3, and protomer 2 or protomer 4. **c,** Topology structure of the Sir2 domain in DSR2. **d,** Structural comparison of the Sir2 domain of ThsA (PDB 6LHX, in gray) and the Sir2 domain of DSR2 (in color). **e,** Structural comparison of the deacetylase Sir2Af1 (PDB 4TWI, in gray) and the Sir2 domain of DSR2 (in color). **f,** Multiple sequence alignment of the conserved catalytic residues in the Sir2 domain of WP_029317421 (*Bacillus subtilis* 29R), WP_010942011.1 (*Geobacter sulfurreducens*), WP_002078322 (*Bacillus cereus* MSX-D12), WP_012679271 (*Streptococcus equi*), Q9WYW0.1 (*Thermotoga maritima*), WP_010877626.1 (*Archaeoglobus fulgidus*), CAI5002430.1 (*Saccharomyces cerevisiae*), and NP_001363727 (*Homo sapiens*). The conserved residues are indicated with red shading. The catalytic residues are indicated with red triangles. **g,** Evolutionary conservation of amino acids of DSR2. Conservations and variations of DSR2 sites are shown in a weblogo plot. The plot was calculated based on multiple sequences alignment (MSA) with 803 homologous sequences. The height of each alphabet in the plot indicates the reduced entropy (conservation) of the amino acid observed in the MSA. The higher the height of the letter, the higher the conservation. The letters on the top of the plot are the amino acids of the DSR2 (8 species) sequences. The diagram below the sequence with red (helix), yellow (sheet) and gray (turn) colors indicates the secondary structures of DSR2. The gray diagram below the secondary structure diagram indicates the solvent accessible surface area (SASA) of the residues; a deeper color indicates a higher value of SASA.

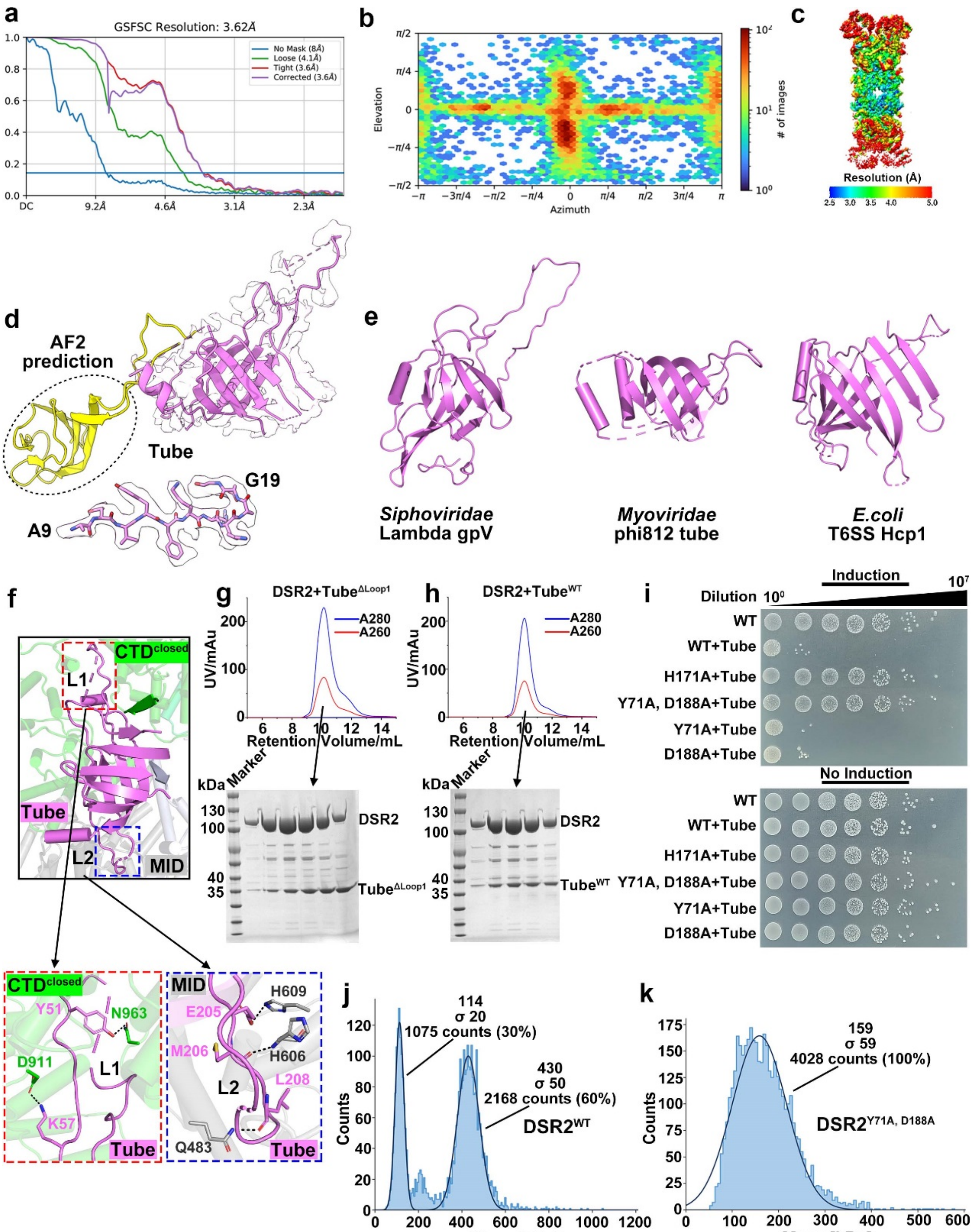

Supplementary Fig. 3: Cryo-EM reconstruction of the DSR2–tube complex.

a, Fourier Shell Correlation curve of the DSR2–tube complex. **b,** Direction distribution plot of the DSR2–tube complex. **c,** Final 3D reconstructed map of the DSR2–tube complex, colored according to local resolution. **d,** Cryo-EM densities of the D2 domain (in violet) of the tube protein of phage SPR bound in the DSR2-tube complex. Densities for the indicated regions are shown in the context of the atomic model. D1 domain (in yellow) was modeled by Alphafold 2. **e,** Structure of the tail tube proteins from classical *Siphoviridae*, *Myoviridae* and *E. coli* T6SS (type VI secretion systems). **f,** The Loop1 (L1) and Loop2 (L2) of the tail tube protein interact with CTD^{closed} and MID domain of

protomer 1, respectively. Expanded views indicate the detailed interactions mediated by L1 and L2. **g and h,** Size exclusion chromatography and SDS-PAGE profiles of the purified DSR2–tube$^{\Delta Loop1}$ (**g**) and DSR2–tube^{WT} (**h**) complexes. **i,** Survival status of *E. coli* cells co-producing DSR2 (WT or variants) and tube. **j and k,** Mass photometry data of WT DSR2 (**j**) and DSR2$^{Y71A,\ D188A}$ (**k**), with masses and respective particle population counts indicated. Source data are provided as a Source Data file.

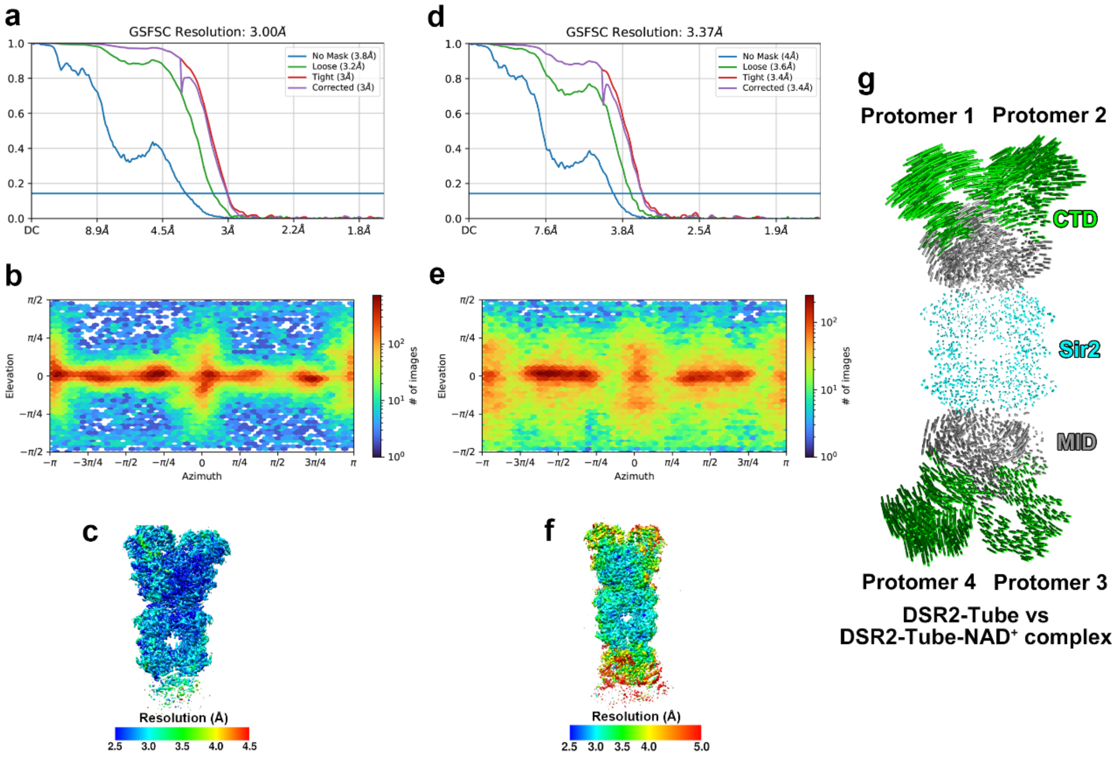

Supplementary Fig. 4: Cryo-EM reconstruction of the DSR2–tube–NAD$^+$ ternary complex.

a, d, Fourier Shell Correlation curve of the DSR2–tube–NAD$^+$ ternary complex. **b, e,** Direction distribution plot of the DSR2–tube–NAD$^+$ ternary complex. **c, f,** Final 3D reconstructed map of the DSR2–tube–NAD$^+$ ternary complex, colored according to local resolution. **g,** Structural comparison between DSR2-tube-NAD$^+$ ternary complex with a DSR2: tube ratio of 4:4 and DSR2–tube complex with a DSR2: tube ratio of 4:2. Vector length correlates with the scale of the domain movement.

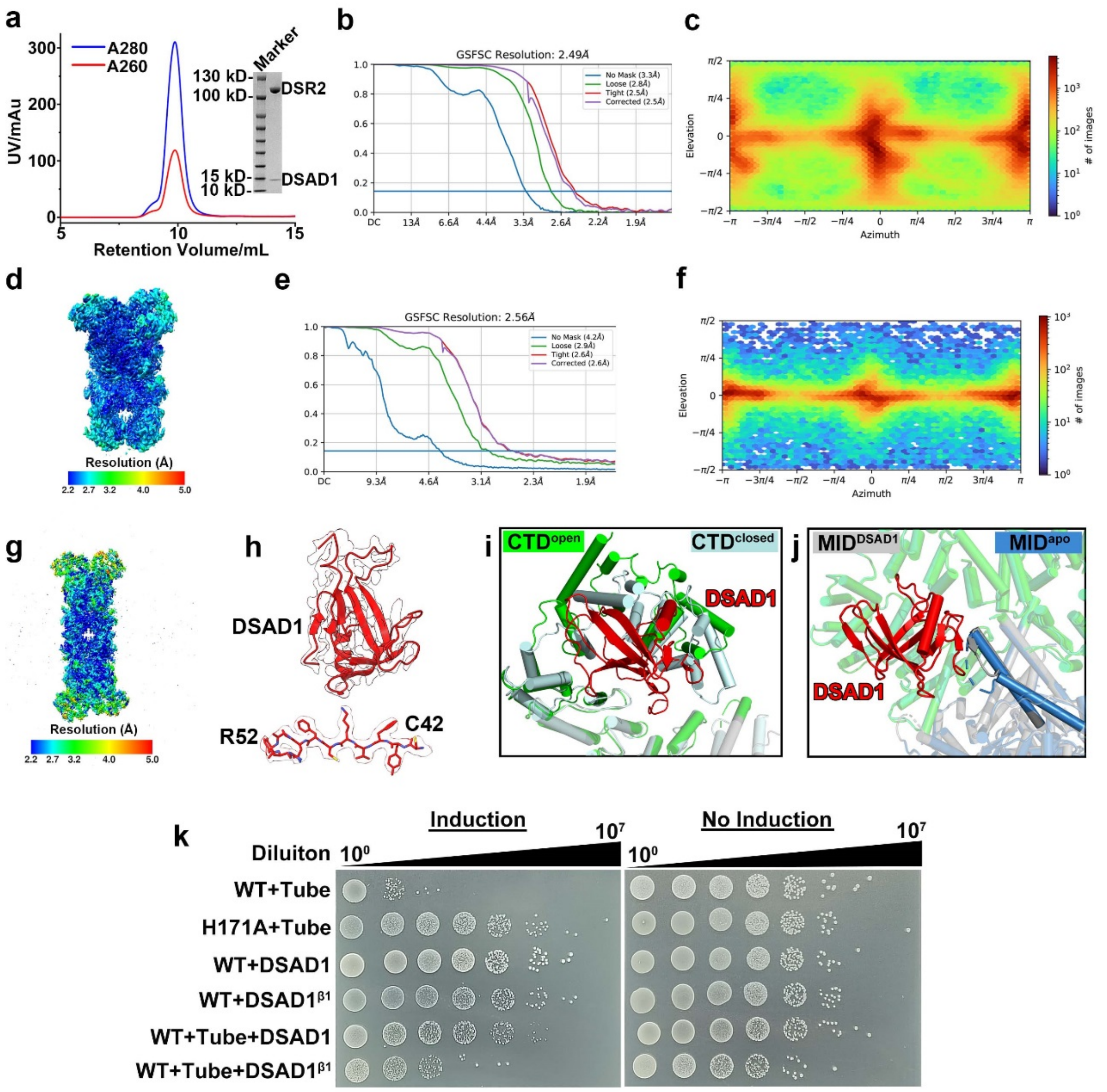

Supplementary Fig. 5: Cryo-EM reconstruction of the DSR2–DSAD1 complex.

a, Size exclusion chromatography and SDS-PAGE profiles of the purified DSR2–DSAD1 complex. **b, e,** Fourier Shell Correlation curve of the DSR2–DSAD1 complex. **c, f,** Direction distribution plot of the DSR2–DSAD1 complex. **d, g,** Final 3D reconstructed map of the DSR2–DSAD1 complex, colored according to local resolution. **h,** Cryo-EM densities of DSAD1 bound in the DSR2–DSAD1 complex. Densities for the indicated regions are shown in the context of the atomic model. **i,** Docking of DSAD1 into CTD^{closed} results in significant clashes. **j,** Structural comparison of MID in the apo form of DSR2 (shown in blue) and in the DSR2–DSAD1 complex (in gray). **k,** Survival status of *E. coli* cells co-producing DSR2 (WT or mutant) and phage tail tube alone or in the presence of DSAD1 (WT or mutant). Source data are provided as a Source Data file.

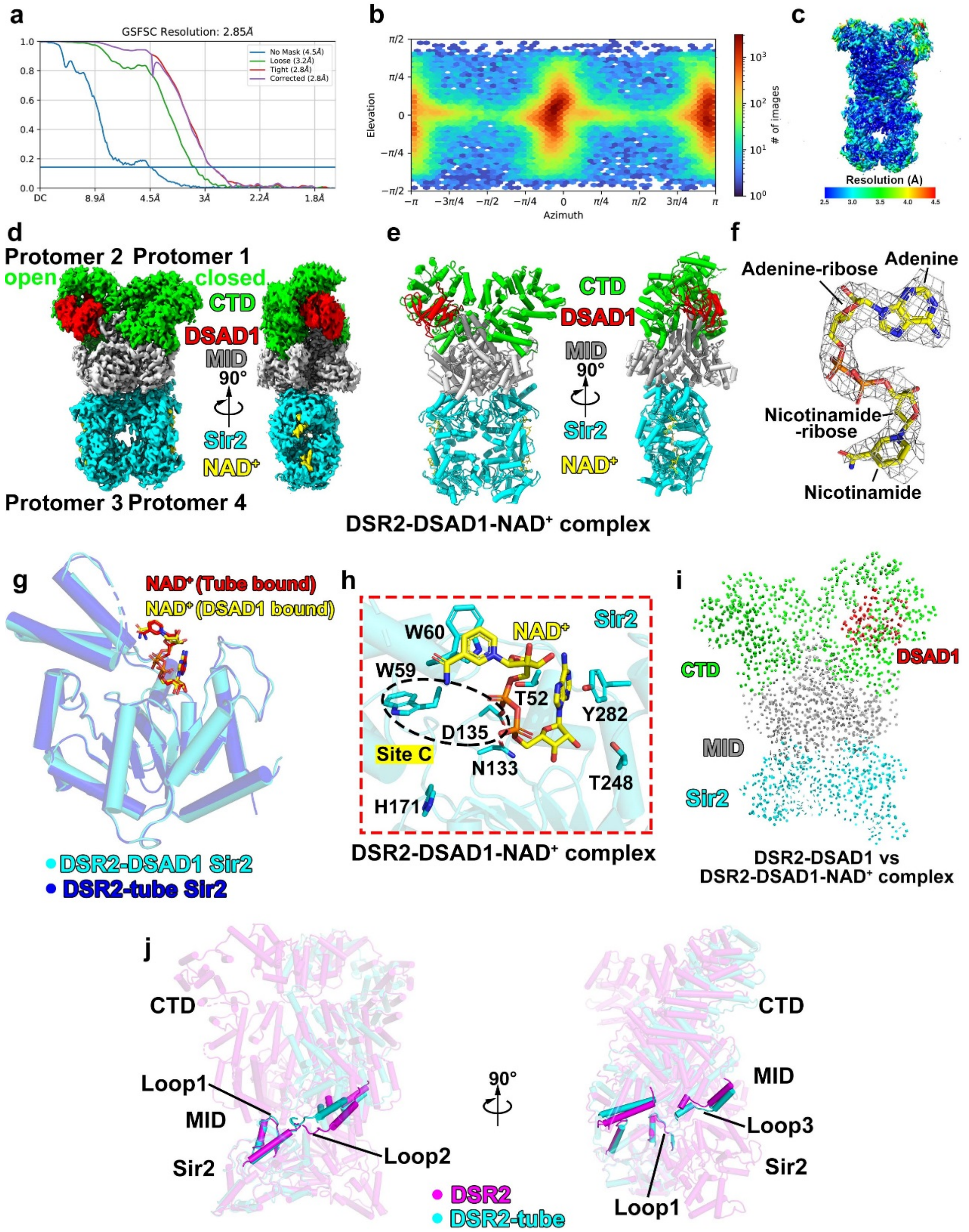

Supplementary Fig. 6: Cryo-EM reconstruction of DSR2–DSAD1–NAD$^+$ ternary complex.

a, Fourier Shell Correlation curve of the DSR2–DSAD1–NAD$^+$ complex. **b,** Direction distribution plot of the DSR2–DSAD1–NAD$^+$ complex. **c,** Final 3D reconstructed map of the DSR2–DSAD1–NAD$^+$ complex, colored according to local resolution. **d, e,** Surface (D) and ribbon (E) representations of the 2.9-Å cryo-EM structure of the DSR2–DSAD1–NAD$^+$ complex. **f,** Electron density map of NAD$^+$ bound in the DSR2–

DSAD1–NAD^+ complex, shown as gray mesh, contoured at 9.0 σ. **g,** Superposition of the Sir2 domain in the DSR2–DSAD1–NAD^+ complex and the DSR2–Tube–NAD^+ complex. **h,** NAD^+ binding pocket in the Sir2 domain within the DSR2–DSAD1–NAD^+ complex. **i,** Structural comparison of the DSR2–DSAD1 binary complex and the DSR2–DSAD1–NAD^+ ternary complex. Vector length correlates with the scale of domain movement. **j,** Structural comparison of the DSR2 complex and the DSR2–Tube binary complex. Three loops located in the Sir2-MID interface with pronounced movements are labeled.

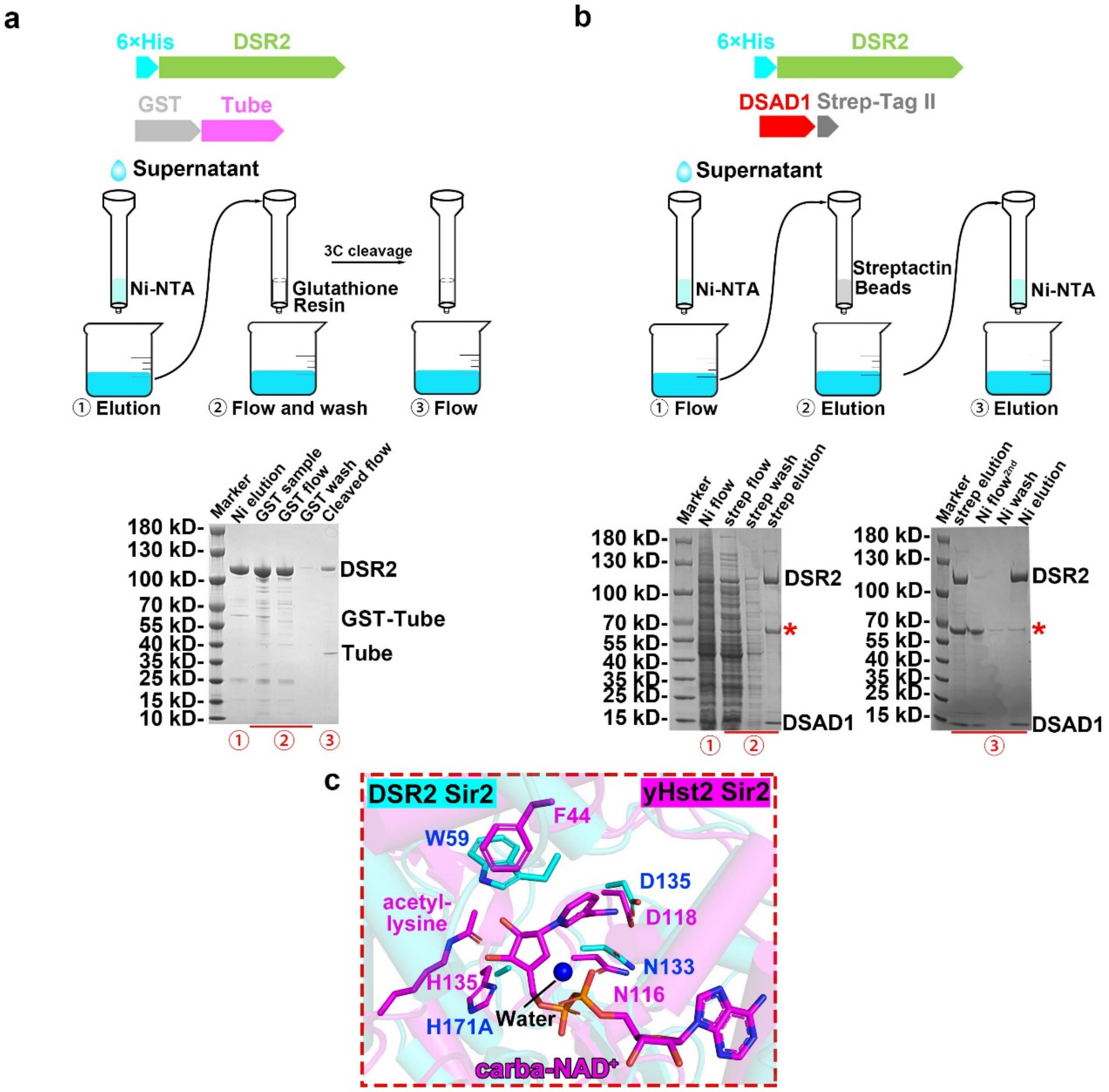

Supplementary Fig. 7: Affinity chromatography analysis reveals excess DSR2 during purification of the DSR2–Tube complex and excess DSAD1 during purification of the DSR2-DSAD1 complex.

a, Flowchart and SDS-PAGE profiles of the purification scheme of the DSR2–tube complex. The supernatant of bacterial cell lysates was loaded onto Ni-NTA resin and the eluate was collected. The eluate was subsequently diluted with buffer A (20 mM Tris-HCl, 100 mM NaCl, 1 mM DTT, pH 7.5) to decrease the imidazole concentration to <20 mM. The sample was then loaded onto glutathione resin. The column was then washed with buffer A. The GST tag was removed by on-column cleavage with HRV 3C protease and the flow-through from cleavage was collected. **b,** Flowchart and SDS-PAGE profiles of purification scheme of the DSR2–DSAD1 complex from Ni-Flow sample. The supernatant of bacterial cell lysates was loaded onto Ni-NTA resin, and the flow-through was loaded onto streptavidin beads. The eluate was then loaded onto Ni-NTA resin and the final eluate was collected. An unknown miscellaneous protein is

indicated by *. **c,** Detailed view of the NAD^+ binding pocket within the Sir2 domain of DSR2 (cyan) and the yHst2 Sir2 deacetylase (PDB: 1SZC) (magenta). The conserved residues and water molecule are indicated. Source data are provided as a Source Data file.

Supplementary Table 1. Cryo-EM data collection, refinement and validation statistics

	DSR2 apo complex	DSR2 apo (partial) complex	DSR2-tube complex	DSR2^{H171A}-tube-NAD$^+$ complex	DSR2^{H171A}-tube-NAD$^+$ (partial) complex	DSR2-DSAD1 complex	DSR2-DSAD1 (partial) complex	DSR2-DSAD1-NAD$^+$ (partial) complex
**Data collection and processing**								
Magnification	105,000	105,000	105,000	105,000	105,000	81,000	105,000	105,000
Voltage (keV)	300	300	300	300	300	300	300	300
Electron exposure (e$^-$/Å^2)	50	50	50	50	50	50	50	50
Defocus range (μm)	1.5 to 2.5	1.5 to 2.5	1.5 to 2.5	1.5 to 2.5	1.5 to 2.5	1.5 to 2.5	1.5 to 2.5	1.5 to 2.5
Pixel size (Å)	0.827	0.827	0.827	0.827	0.827	0.988	0.827	0.827
Symmetry imposed	C1	C1	C1	C1	C1	C1	C1	C1
Initial particle images (no.)	1,631,393	1,631,393	454,453	936,112	302,075	3,172,644	3,172,644	2,452,020
Final particle images (no.)	59,543	364,318	16,411	67,895	85,495	101,402	946,024	230,498
Map resolution (Å)	3.10	2.57	3.59	3.35	2.97	2.57	2.47	2.82
FSC threshold	0.143	0.143	0.143	0.143	0.143	0.143	0.143	0.143
Map resolution range (Å)	2.2~999	2.2~999	2.2~999	2.2~999	2.2~999	2.2~999	2.2~999	2.2~999
**Refinement**								
Initial model used (PDB code)	*ab-initio*	*ab-initio*	*ab-initio*	*ab-initio*	*ab-initio*	*ab-initio*	*ab-initio*	*ab-initio*
Model resolution (Å)	3.37	2.73	4.05	3.60	3.17	3.13	2.75	3.06
FSC threshold	0.5	0.5	0.5	0.5	0.5	0.5	0.5	0.5
Map sharpening *B* factor (Å^2)	-90.0	-76.4	-41.3	-82.0	-77.9	-47.1	-107.8	-83.7
Map Correlation Coefficient	0.86	0.86	0.79	0.75	0.85	0.85	0.86	0.86
Model composition								
Non-hydrogen atoms	31,412	20,129	32,639	35,679	22,493	33,765	21,232	21,512
Protein residues	3,757	2,419	3,912	4,270	2,681	4,043	2,553	2,553
Ligands	0	0	0	4	4	0	0	4
B factor (Å^2)								
Protein	195.24	61.00	241.99	55.14	47.09	96.59	24.04	91.31
Ligand				52.08	76.40			104.64
R.m.s. deviations								
Bond lengths (Å)	0.002	0.002	0.003	0.003	0.002	0.003	0.003	0.003
Bond angles (°)	0.552	0.508	0.710	0.685	0.459	0.577	0.517	0.421
Validation								
MolProbity score	1.63	1.92	2.10	1.90	2.85	1.91	1.96	2.46
Clash score	12.27	9.74	16.09	15.79	37.82	11.86	9.52	34.30
Poor rotamers (%)	0.14	3.47	1.67	0.49	6.55	2.63	9.52	3.24
Ramachandran plot								
Favored (%)	97.86	98.24	96.54	96.81	96.99	98.19	98.17	97.78
Allowed (%)	2.14	1.76	3.46	3.19	3.01	1.81	1.83	2.22
Disallowed (%)	0.00	0.00	0.00	0.00	0.00	0.00	0.00	0.00
EMDB	37919	37920	37921	37922	37923	37924	37925	37926
PDB	8WY8	8WY9	8WYA	8WYB	8WYC	8WYD	8WYE	8WYF

Source Data Supplementary Fig. 1

Figure S1a

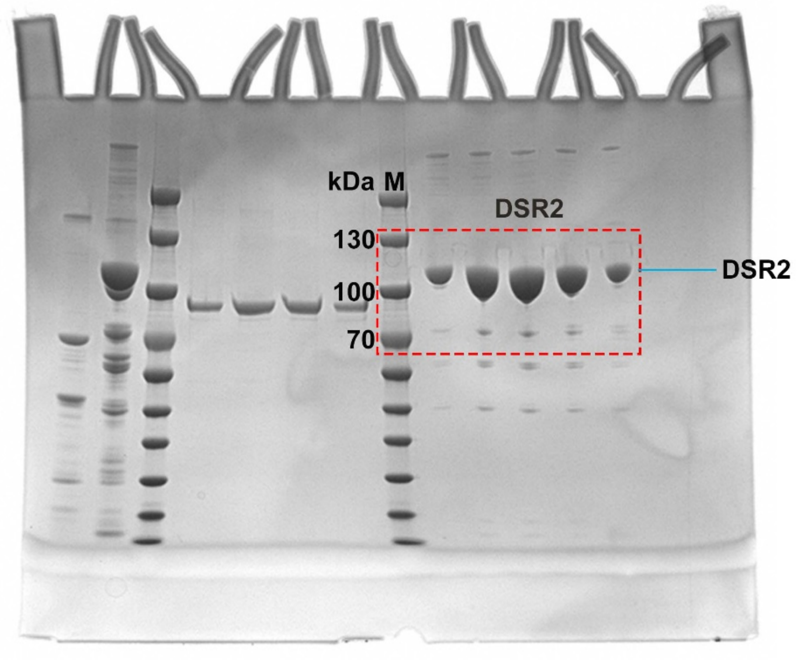

Source Data Supplementary Fig. 3

Figure S3g,h

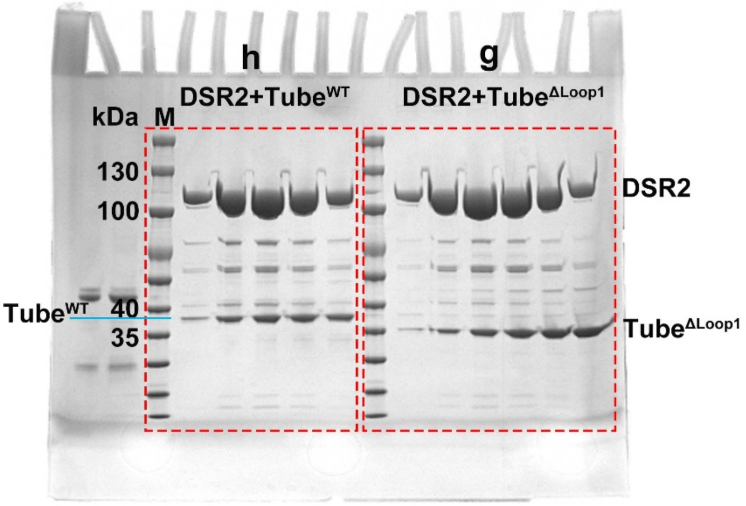

Figure S3i

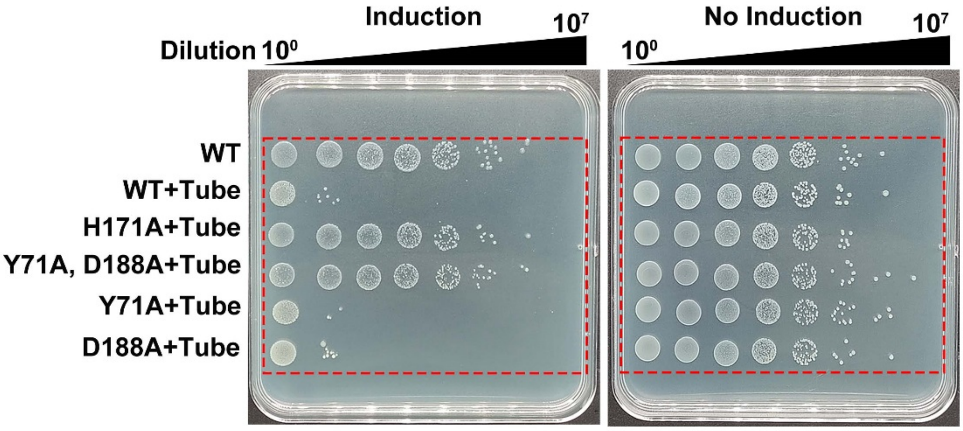

Source Data Supplementary Fig. 5

Figure S5a

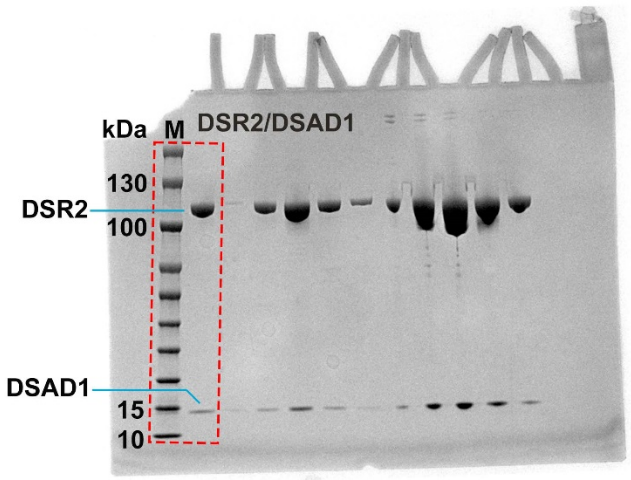

Figure S5k

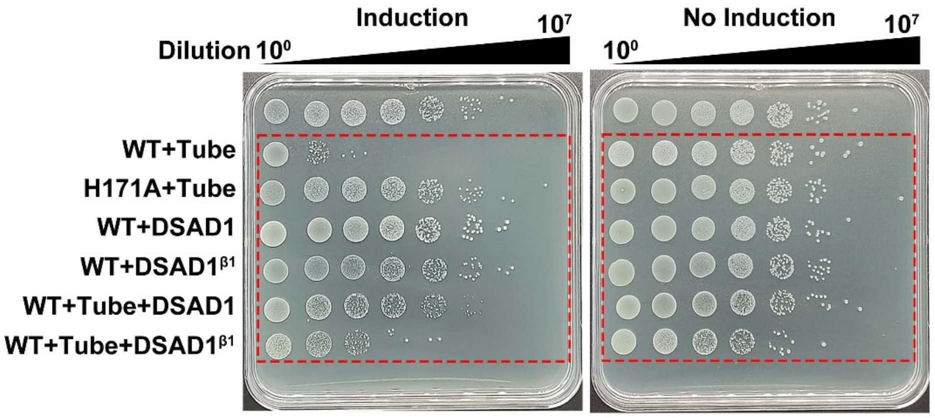

Source Data Supplementary Fig. 7

Figure S7a

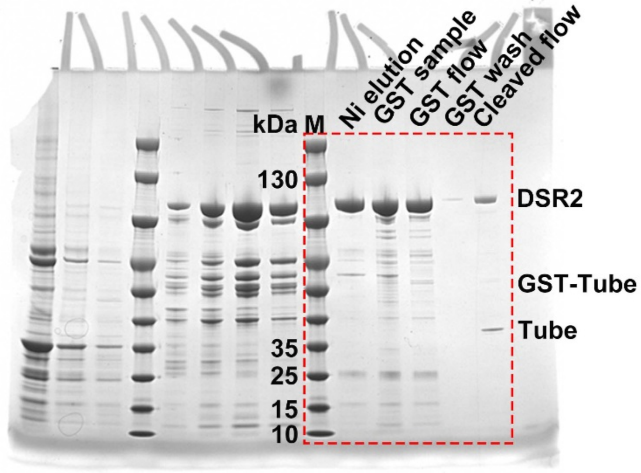

Figure S7b

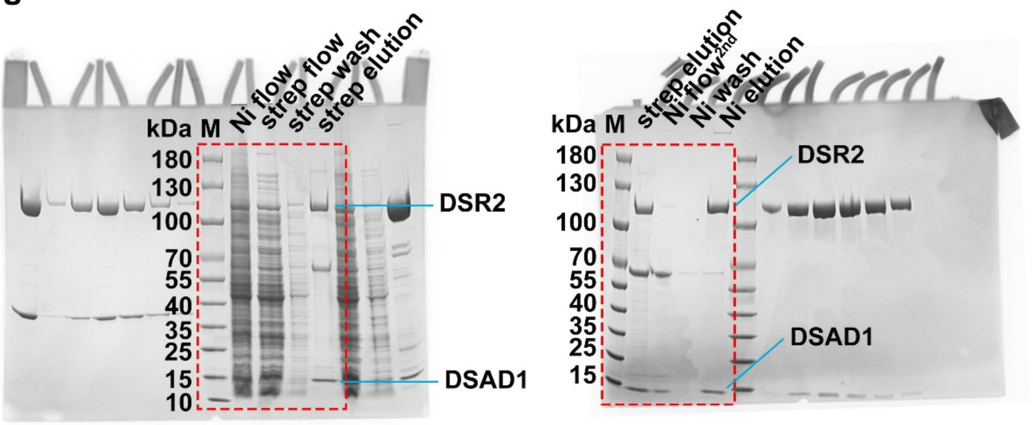

Supplement: Supplementary file 1 — Supplementary Information [file 41467_2024_47177_MOESM1_ESM.pdf]
