# Supplementary material for: Structural basis for phage-mediated activation and repression of bacterial DSR2 anti-phage defense system
Source: Nat Commun. 2024 Mar 30;15:2797. doi: 10.1038/s41467-024-47177-9 (PMC10981675; doi:10.1038/s41467-024-47177-9)
Supplement: Supplementary file 3 — Description of Additional Supplementary Files [file 41467_2024_47177_MOESM3_ESM.pdf]

File name: Supplementary Data 1

Description: Primers used in this study.
